# Expanding Horizons: Re-Viewing Aging and the Arts

**DOI:** 10.1093/geroni/igaf122.1676

**Published:** 2025-12-31

**Authors:** Justine McGovern, James Powers, Desmond O’Neill

## Abstract

Across four presentations, this symposium explores new horizons in gerontology in terms of content and format. Not only does it examine representations of old age in the arts and the role of engaging in the arts into later life from a research perspective, but also it provides a platform for older artists to discuss their visual practice and introduce their work to new audiences. By blurring the lines between gerontology research and arts practice, the symposium challenges ingrained hierarchies and categories of knowledge production and dissemination, and belonging. As a result, the symposium is in itself an example of innovative gerontology. The presentations encourage the audience to seek creative ways of generating knowledge that combine traditional research, gerontology practice, and the arts. Moreover, including the voices of older artists puts their practice on equal footing with research in knowledge-building processes. Takeaways from the session include a deepened understanding of the impact of arts engagement across the life span, increased awareness of how to generate gerontology knowledge in new ways, and an improved outlook on the possibilities of later life.

